# Supplementary material for: Impact of liver-specific survival motor neuron (SMN) depletion on central nervous system and peripheral tissue pathology
Source: eLife. 2025 Feb 20;13:RP99141. doi: 10.7554/eLife.99141 (PMC11841985; doi:10.7554/eLife.99141)
Supplement: Figure 1—source data 1. [file elife-99141-fig1-data1.pdf]

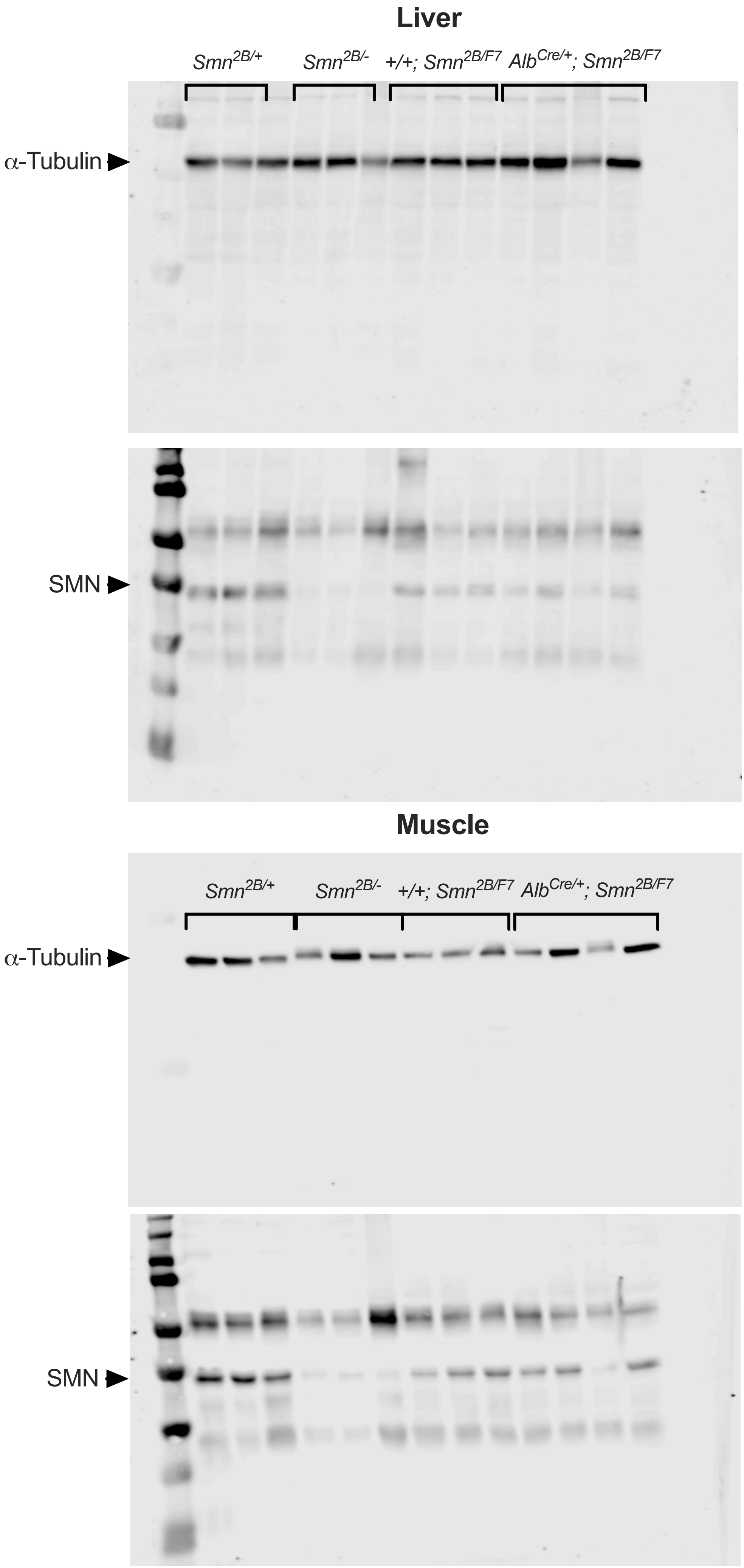
Liver
Smn2B/+
Smn2B/-
+/+; Smn2B/F7
AlbCre/+; Smn2B/F7
α-Tubulin
SMN
Muscle
Smn2B/+
Smn2B/-
+/+; Smn2B/F7
AlbCre/+; Smn2B/F7
α-Tubulin
SMN

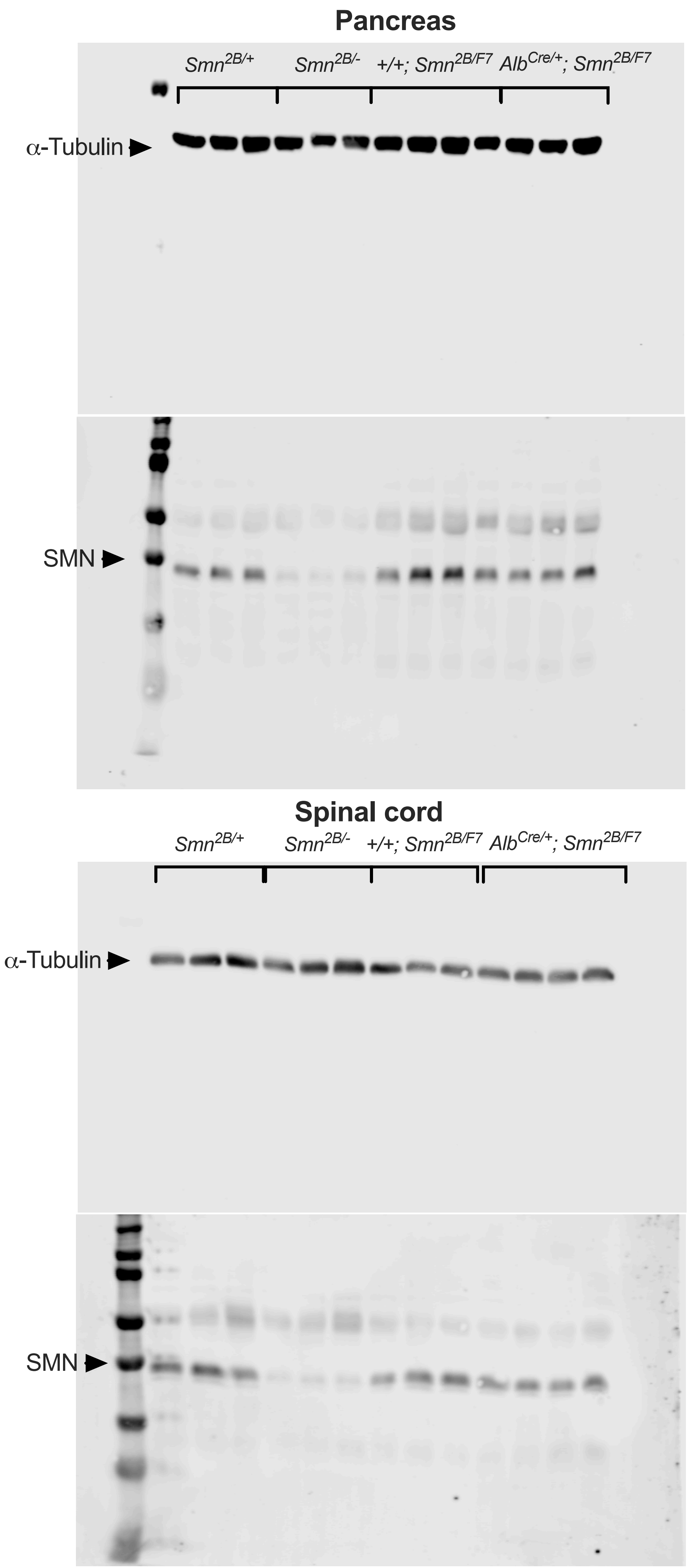
Pancreas
$Smn^{2B/+}$
$Smn^{2B/-}$
+/+; $Smn^{2B/F7}$
$Alb^{Cre/+}$; $Smn^{2B/F7}$
α-Tubulin
SMN
Spinal cord
$Smn^{2B/+}$
$Smn^{2B/-}$
+/+; $Smn^{2B/F7}$
$Alb^{Cre/+}$; $Smn^{2B/F7}$
α-Tubulin
SMN

Brain

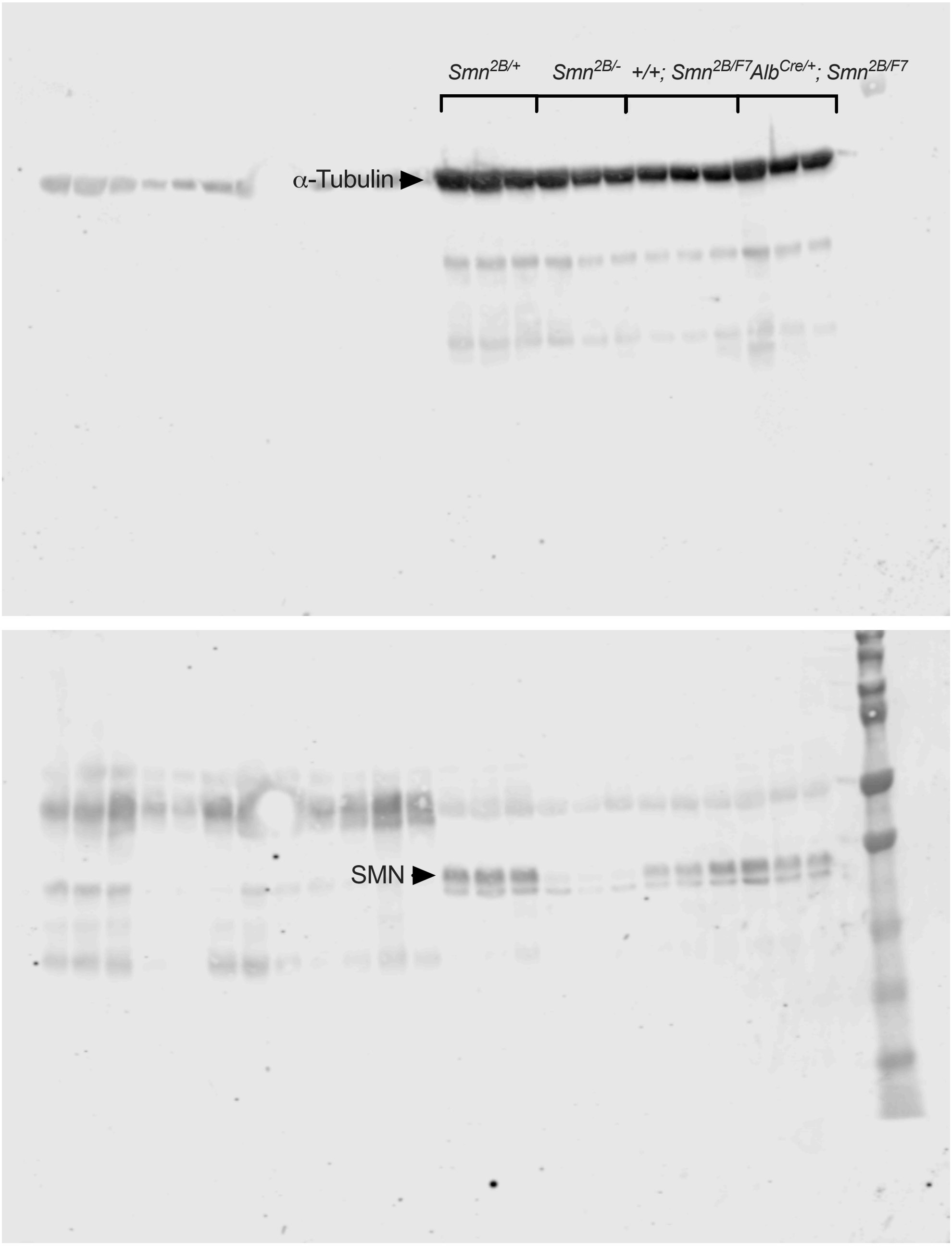

Figure 1- Source Data 1. Original membranes corresponding to Figure 1, panels D-H. Molecular weight markers were employed. The membranes correspond to each tissue separately (liver, brain, skeletal muscle, spinal cord, and pancreas) for tubulin and SMN.
