# Supplementary figures and images for: Impact of liver-specific survival motor neuron (SMN) depletion on central nervous system and peripheral tissue pathology
Source: eLife. 2025 Feb 20;13:RP99141. doi: 10.7554/eLife.99141 (PMC11841985; doi:10.7554/eLife.99141)

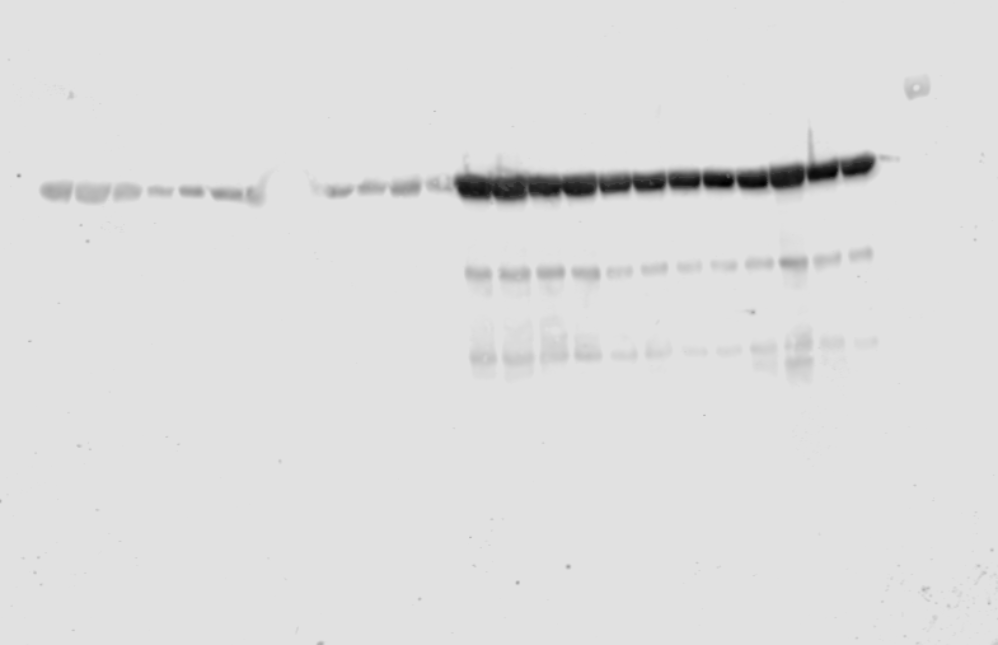

Supplement: Figure 1—source data 2. [file elife-99141-fig1-data2.zip › Figure 1-Source data 2/SMN Brain/Tubulin 50kDa_Brain.tif]

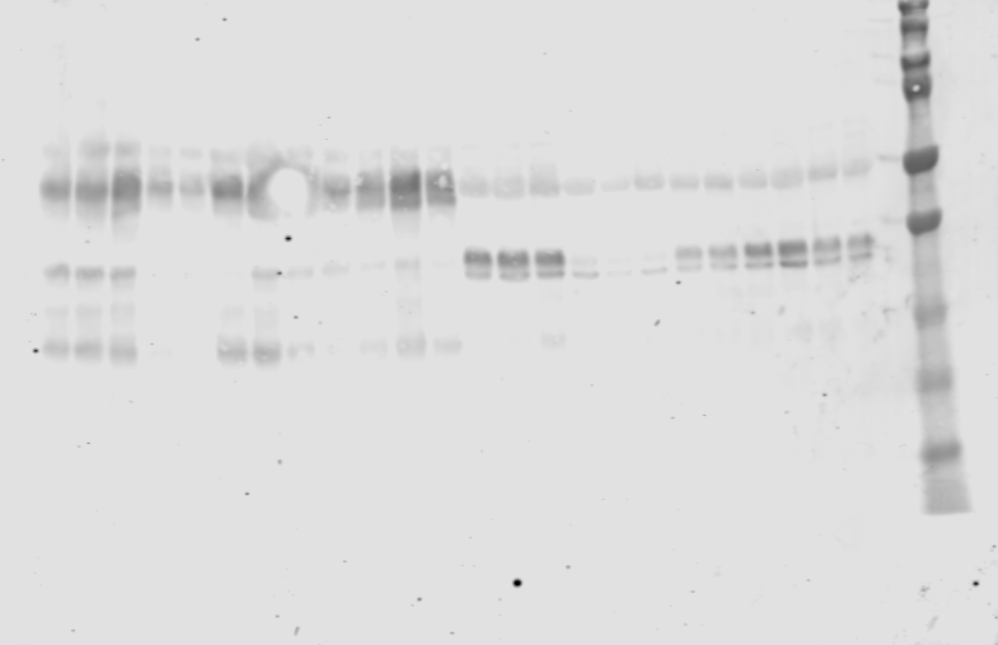

Supplement: Figure 1—source data 2. [file elife-99141-fig1-data2.zip › Figure 1-Source data 2/SMN Brain/SMN ~40kDa_Brain.tif]

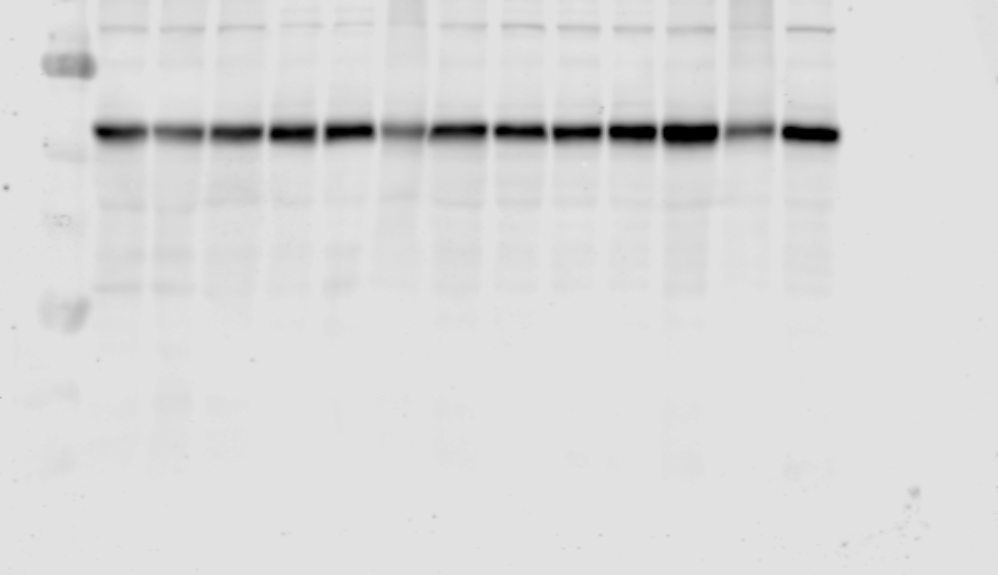

Supplement: Figure 1—source data 2. [file elife-99141-fig1-data2.zip › Figure 1-Source data 2/SMN Liver/Tubulin 50kDa_Liver.tif]

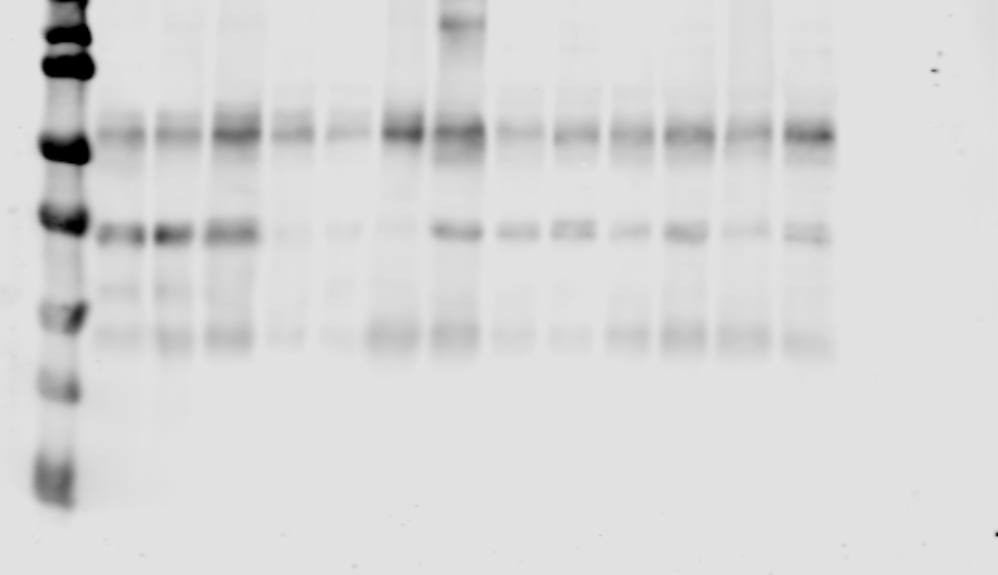

Supplement: Figure 1—source data 2. [file elife-99141-fig1-data2.zip › Figure 1-Source data 2/SMN Liver/SMN ~40kDa_Liver.tif]

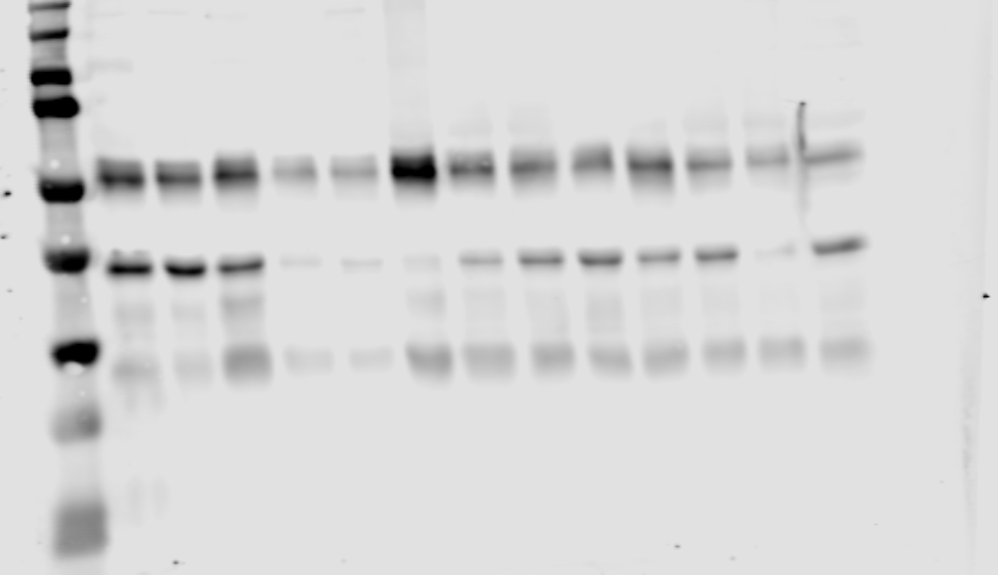

Supplement: Figure 1—source data 2. [file elife-99141-fig1-data2.zip › Figure 1-Source data 2/SMN Muscle/SMN ~40kDa_Muscle.tif]

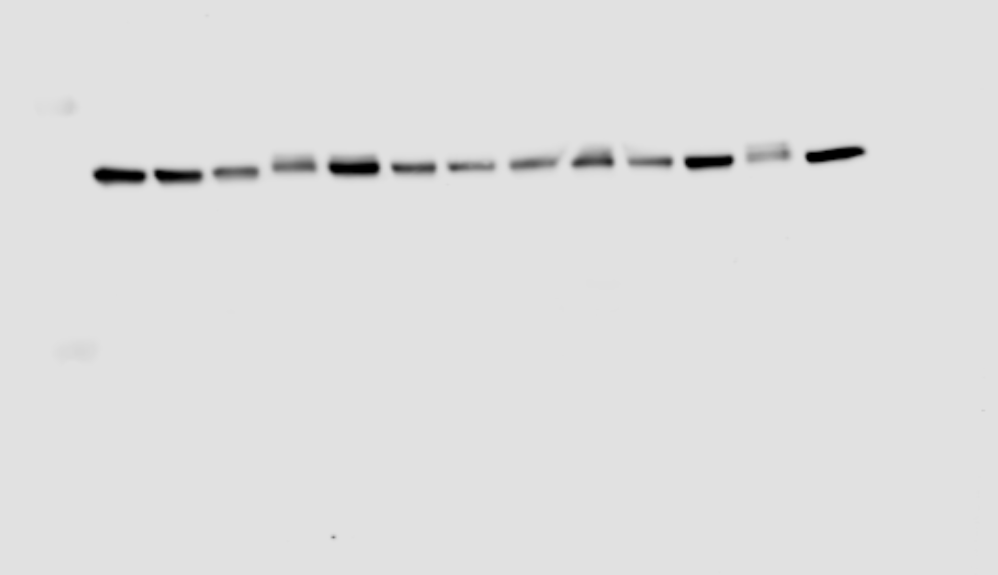

Supplement: Figure 1—source data 2. [file elife-99141-fig1-data2.zip › Figure 1-Source data 2/SMN Muscle/Tubulin 50kDa_Muscle.tif]

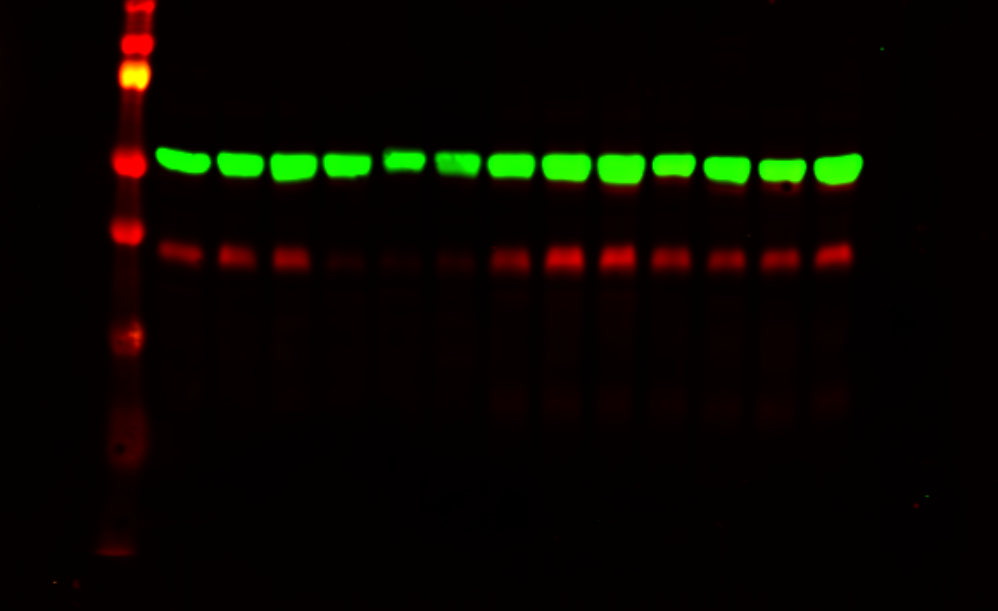

Supplement: Figure 1—source data 2. [file elife-99141-fig1-data2.zip › Figure 1-Source data 2/SMN Pancreas/SMN_red Tubulin_green - Pancreas.tif]

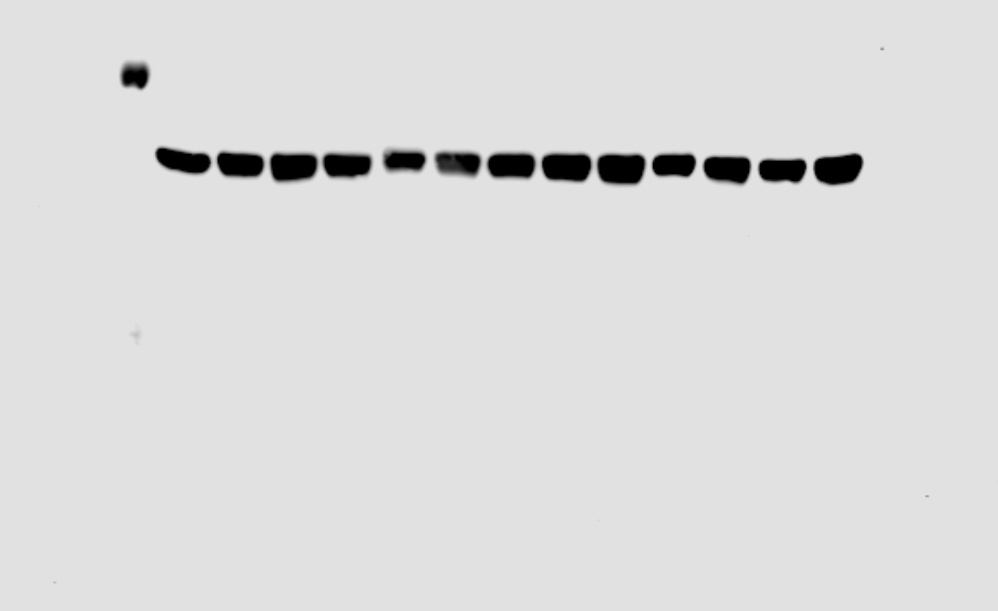

Supplement: Figure 1—source data 2. [file elife-99141-fig1-data2.zip › Figure 1-Source data 2/SMN Pancreas/Tubulin 50kDa - Pancreas.tif]

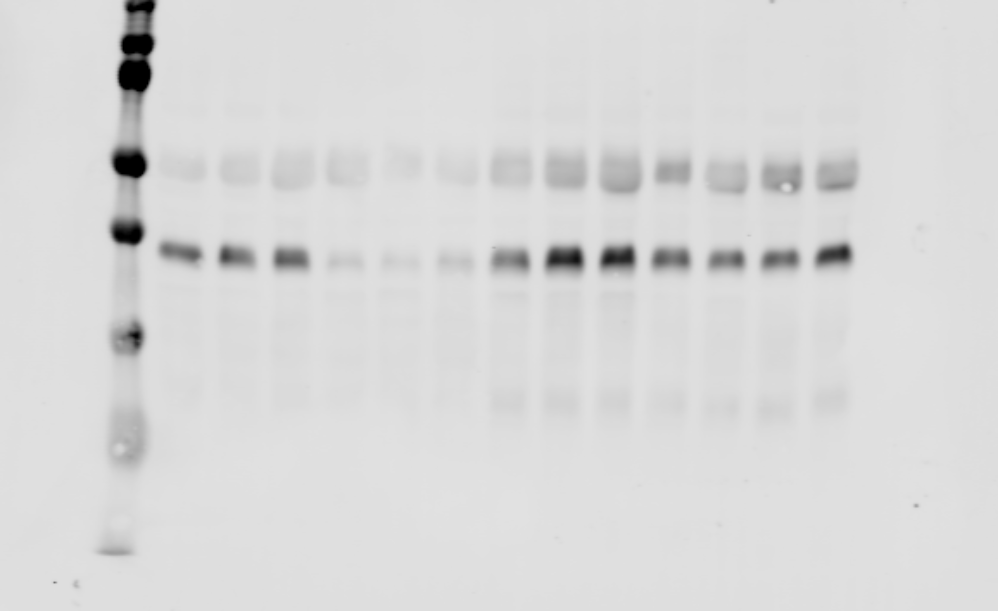

Supplement: Figure 1—source data 2. [file elife-99141-fig1-data2.zip › Figure 1-Source data 2/SMN Pancreas/SMN ~40kDa_Pancreas .tif]

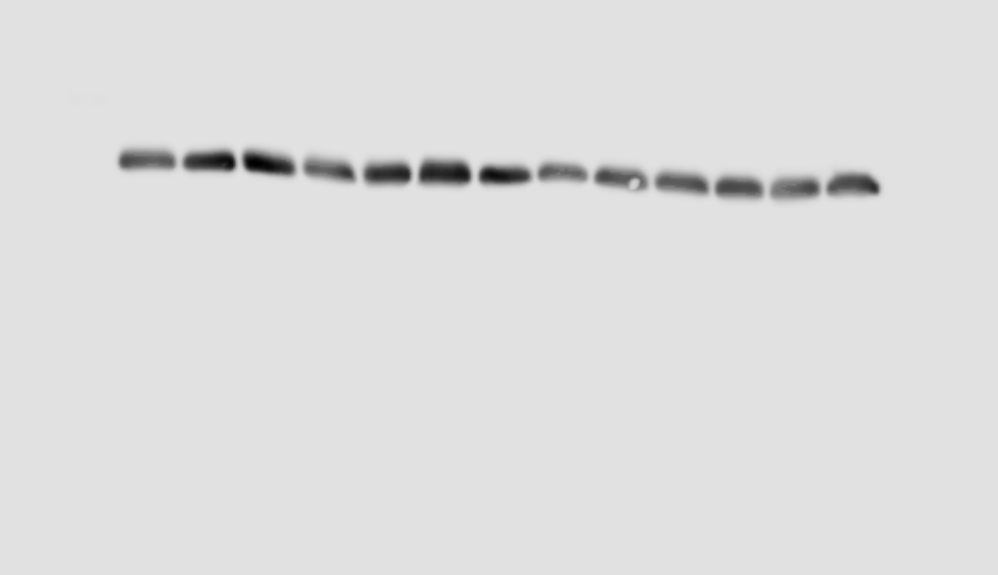

Supplement: Figure 1—source data 2. [file elife-99141-fig1-data2.zip › Figure 1-Source data 2/SMN SC/Tubulin 50kDa_SC.tif]

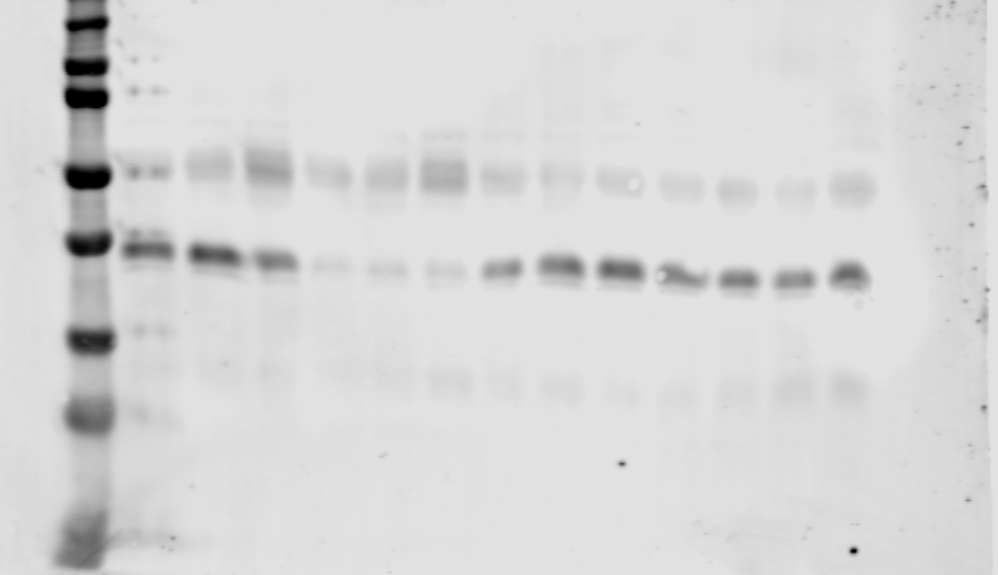

Supplement: Figure 1—source data 2. [file elife-99141-fig1-data2.zip › Figure 1-Source data 2/SMN SC/SMN ~40kDa_SC.tif]

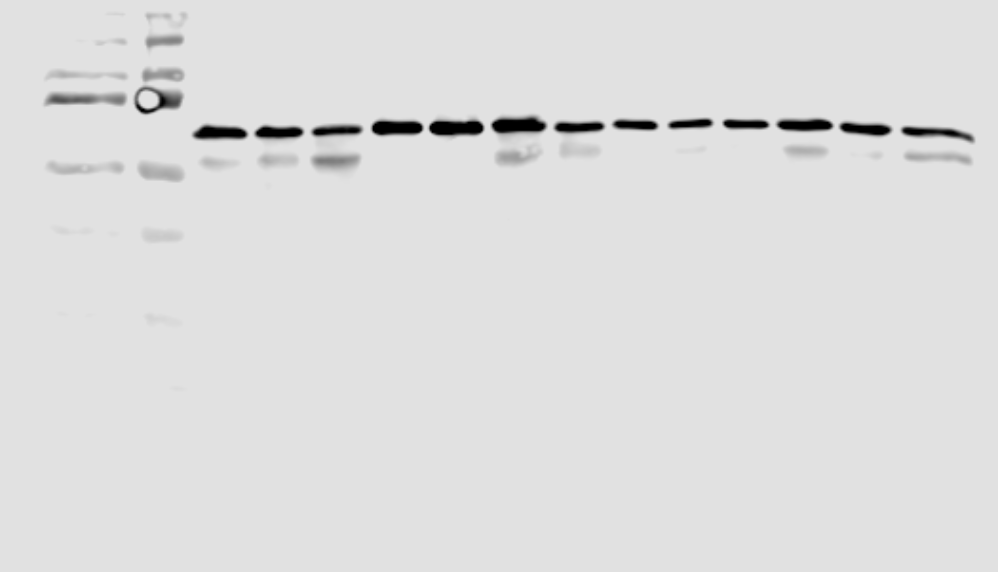

Supplement: Figure 2—source data 2. [file elife-99141-fig2-data2.zip › Figure 2-Source data 2/P62 ~62kDa/P62 ~62kDa.tif]

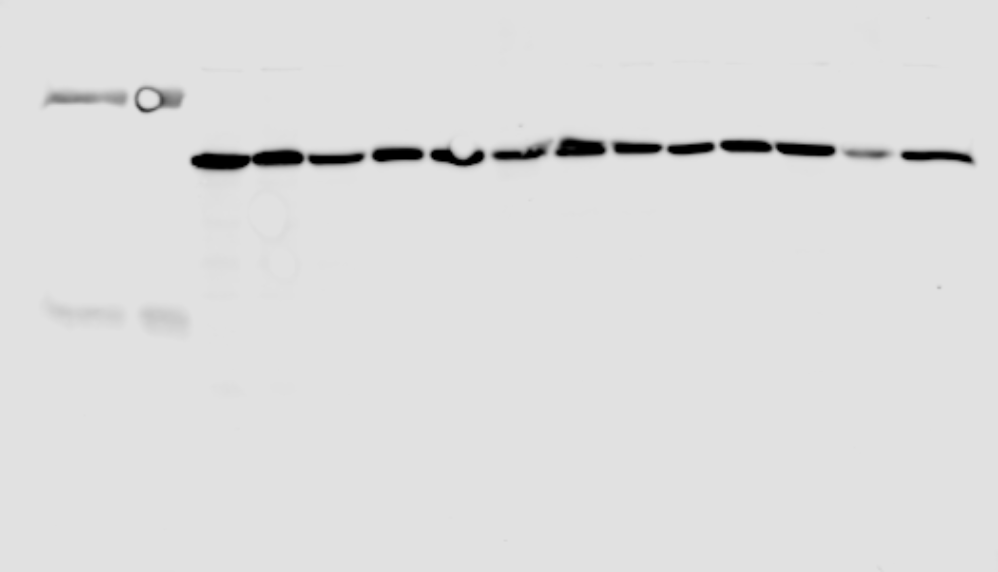

Supplement: Figure 2—source data 2. [file elife-99141-fig2-data2.zip › Figure 2-Source data 2/P62 ~62kDa/Tubulin 50kDa.tif]

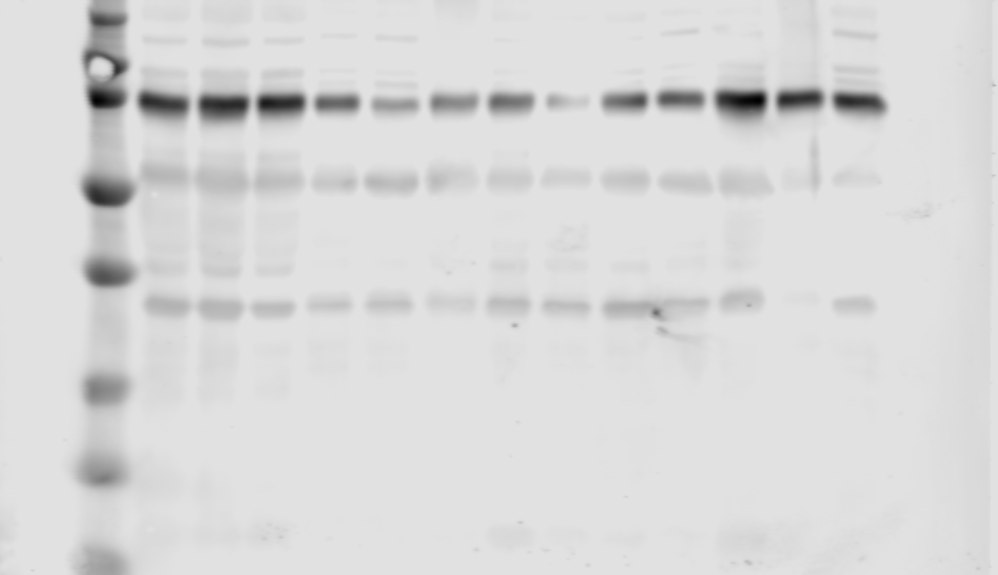

Supplement: Figure 2—source data 2. [file elife-99141-fig2-data2.zip › Figure 2-Source data 2/Transferrin ~77kDa/Transferrin ~77 kDa.tif]

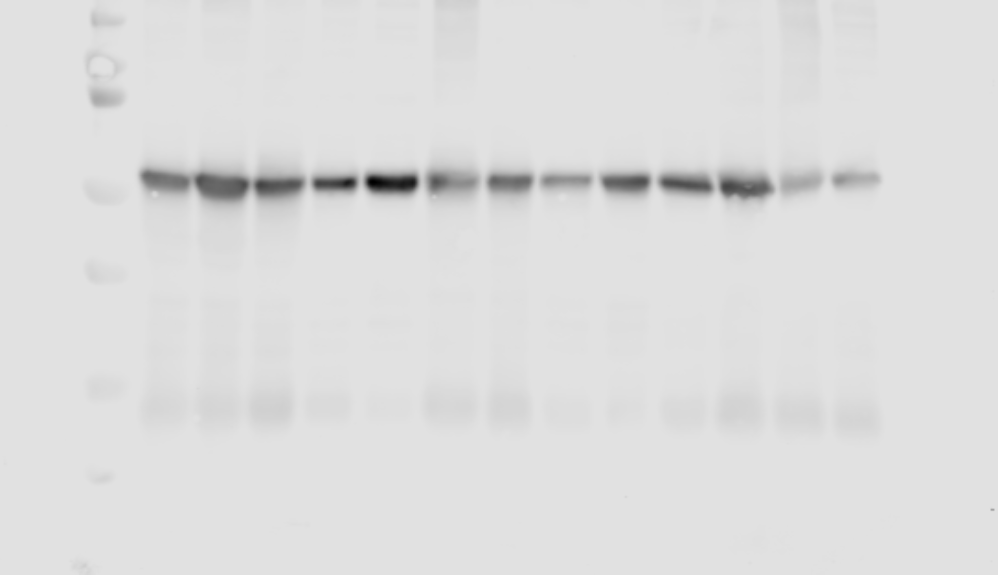

Supplement: Figure 2—source data 2. [file elife-99141-fig2-data2.zip › Figure 2-Source data 2/Transferrin ~77kDa/Tubulin-50kDa.tif]

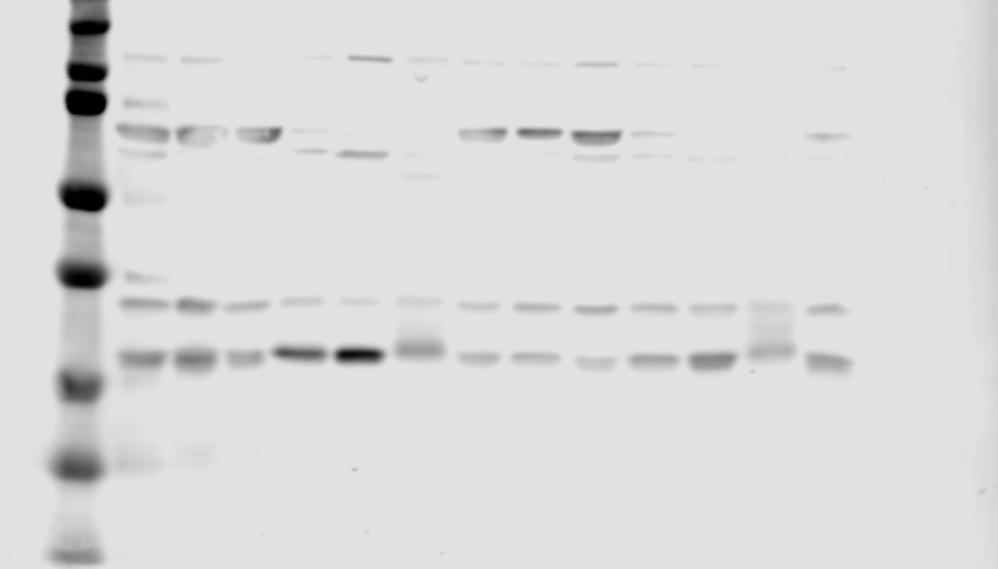

Supplement: Figure 2—source data 2. [file elife-99141-fig2-data2.zip › Figure 2-Source data 2/Heme Oxygenase ~33kDa/Heme Oxygenase ~33kDa.tif]

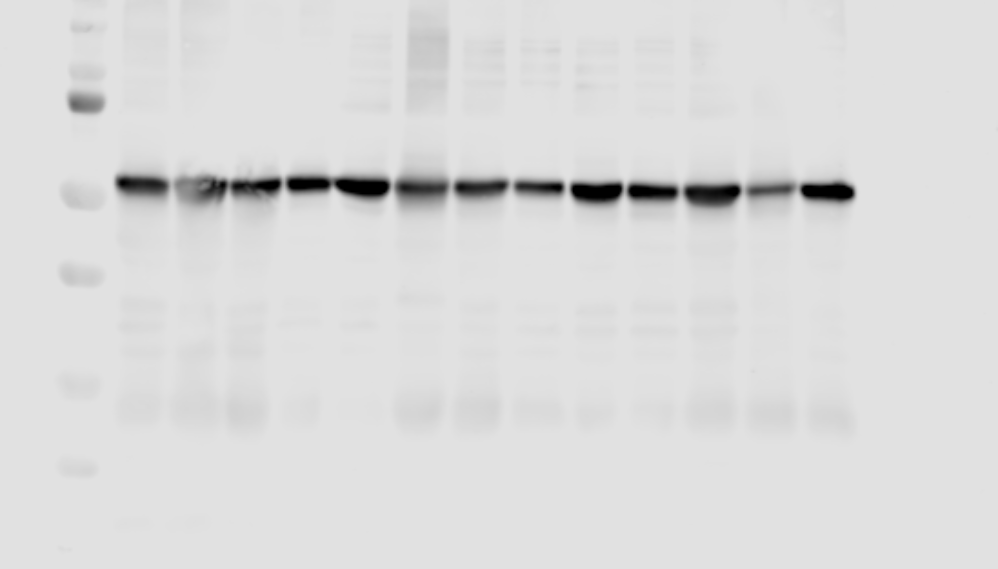

Supplement: Figure 2—source data 2. [file elife-99141-fig2-data2.zip › Figure 2-Source data 2/Heme Oxygenase ~33kDa/Tubulin 50kDa.tif]
