# Supplementary material for: Impact of liver-specific survival motor neuron (SMN) depletion on central nervous system and peripheral tissue pathology
Source: eLife. 2025 Feb 20;13:RP99141. doi: 10.7554/eLife.99141 (PMC11841985; doi:10.7554/eLife.99141)
Supplement: Figure 2—source data 1. [file elife-99141-fig2-data1.pdf]

Heme oxygenase (HO)

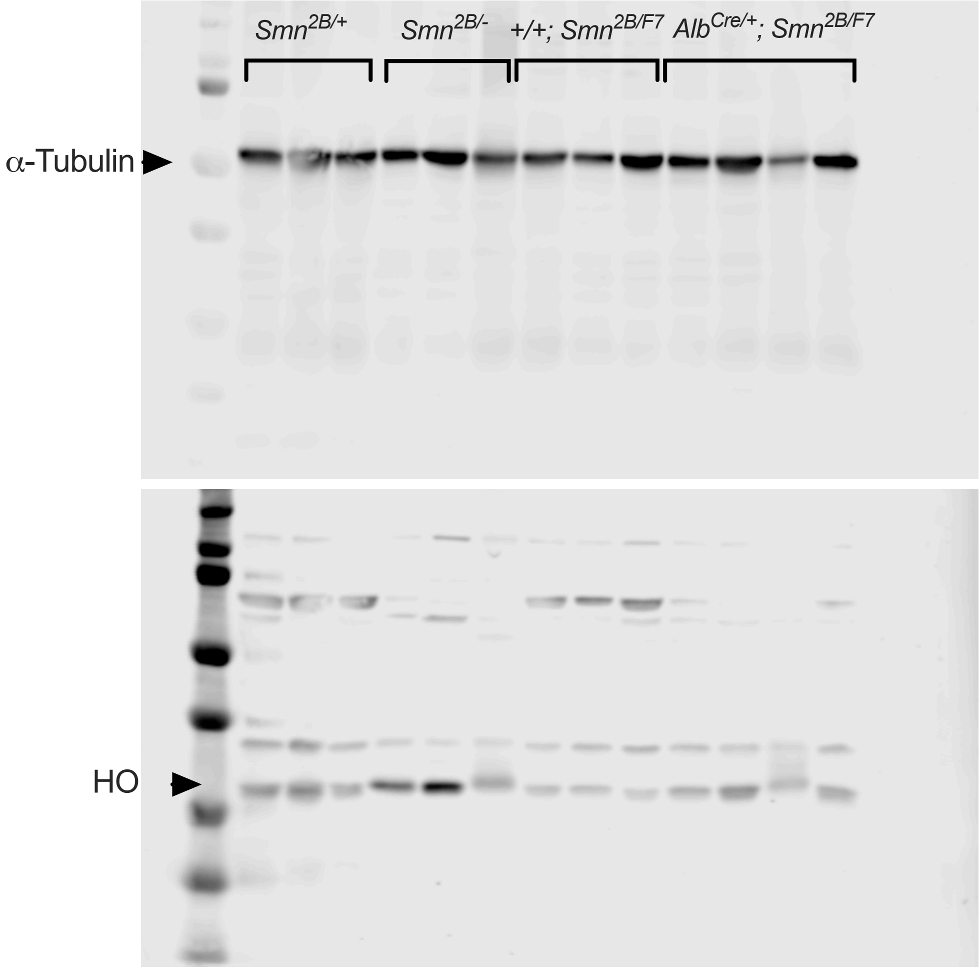

P62

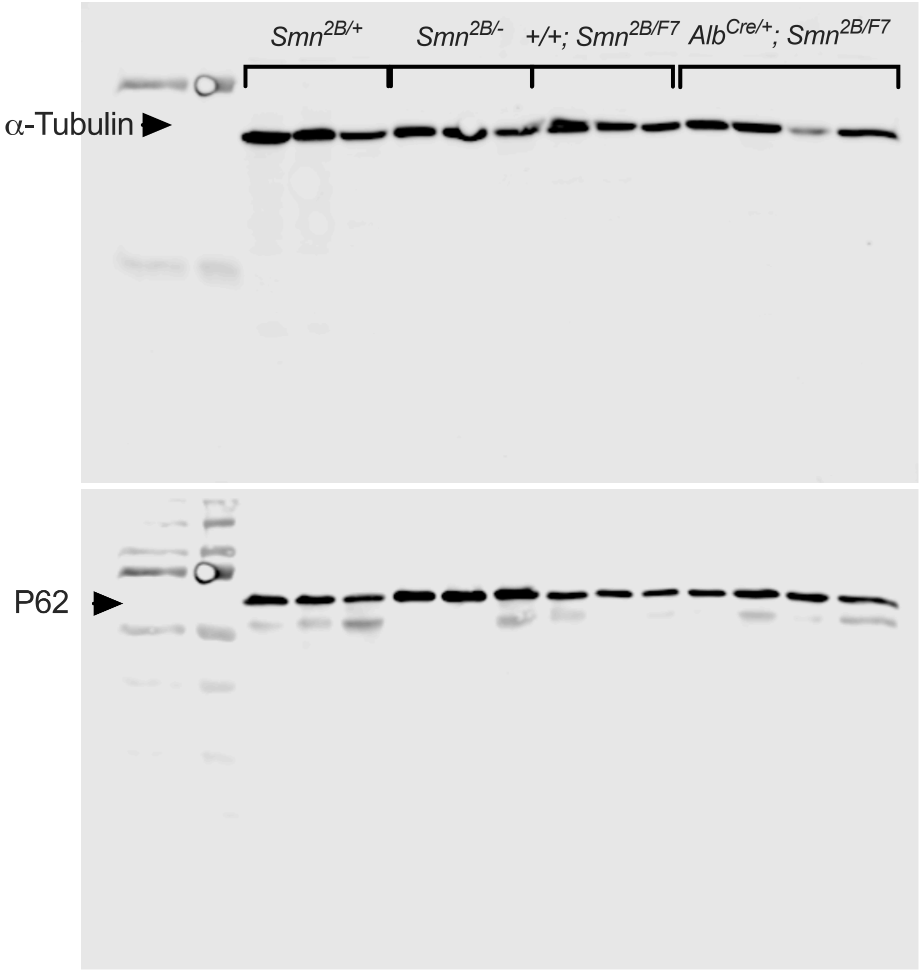

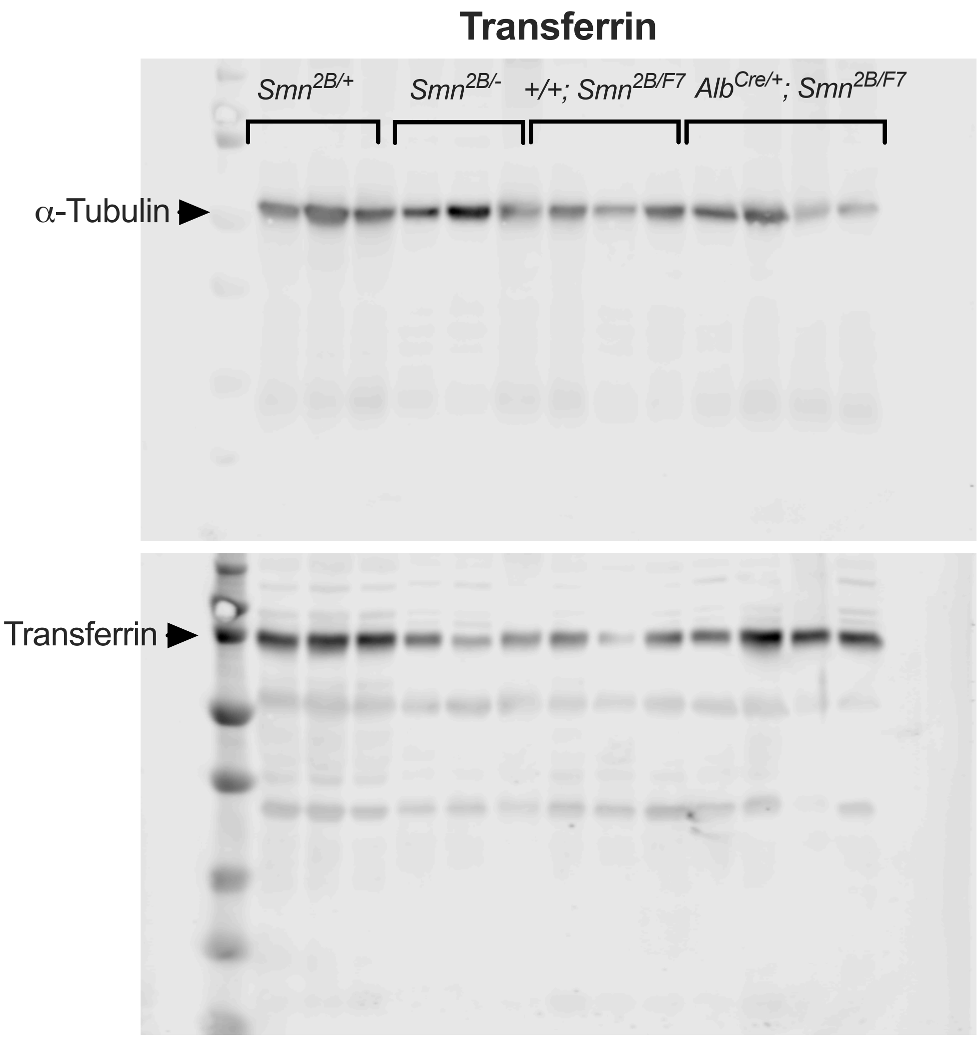
Transferrin
Smn2B/+
Smn2B/-
+/+; Smn2B/F7
AlbCre/+; Smn2B/F7
α-Tubulin
Transferrin

Figure 2 - Source Data 1. Original membranes corresponding to Figure 2, panels C-E. Molecular weight markers were employed. The membranes correspond to Heme oxygenase, P62, and transferrin separately. The blots for the tubulin controls are also shown.
